# Piezo2 Mediates a Vicious Cycle of “Mechanical Homeostasis Imbalance—Inflammation” in Sensory Nerves and the Cartilage Endplate

**DOI:** 10.1002/advs.202507299

**Published:** 2026-04-16

**Authors:** Hanpeng Xu, Wen Geng, Zhi Du, Yifan Du, Di Wu, Bide Tong, Huaizhen Liang, Xingyu Zhou, Zixuan Ou, Junyu Wei, Kun Wang, Yu Song, Wenbin Hua, Yan Xu, Wencan Ke, Bingjin Wang, Cao Yang

**Affiliations:** ^1^ Department of Orthopaedics Union Hospital Tongji Medical College Huazhong University of Science and Technology Wuhan China; ^2^ Department of Ophthalmology Union Hospital Tongji Medical College Huazhong University of Science and Technology Wuhan China; ^3^ Shenzhen Huazhong University of Science and Technology Research Institute Shenzhen China

**Keywords:** cartilage endplate, intervertebral disk degeneration, low back pain, Piezo2, sensory nerve

## Abstract

Low back pain is a global health problem. Discogenic low back pain, the most common type of low back pain, is closely related to cartilage endplate (CEP) degeneration and inflammation. In this study, by constructing a rat model of lumbar spine instability (LSI) and combining biomechanical analysis with molecular biology techniques, we revealed the central role of the mechanosensitive channel Piezo2 in the vicious cycle of discogenic low back pain. The results revealed that abnormal mechanical stress triggers calcium influx and promotes CGRP release by activating Piezo2 in the dorsal root ganglion (DRG). CGRP synergizes with mechanical force to activate the IKKβ/NF‐κB pathway in CEP cells via the receptor RAMP1, which induces the secretion of IL‐6 and IL‐1β, and further sensitizes DRG neurons, forming a positive feedback loop. Macrophage depletion did not alleviate pain/inflammation. Targeted inhibition of Piezo2 (with AAV‐shPiezo2 gene silencing or omega‐3 fatty acids) or blockade of CGRP signaling (with Rimegepant) significantly alleviated pain‐related behaviors, suppressed inflammation, and delayed CEP degeneration. This study elucidated a novel mechanism by which mechanical‒neuroinflammatory interactions drive the progression of discogenic low back pain and provided a theoretical basis for the development of multitargeted combination treatment strategies.

## Graphical abstract

In a lumbar spine instability model, dorsal root ganglion cells mediate the perception of relevant mechanical stresses through Piezo2 and subsequently release CGRP. CGRP activates the NF‐κB signaling pathway in cartilage endplate cells through the receptor RAMP1. This results in the release of IL‐6 and IL‐1β and the sensitization of neuronal cells, creating a positive feedback loop.

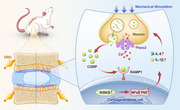

## 1 Introduction

Low back pain is a globally recognized healthcare challenge that affects people's activities of daily life and productivity and imposes a serious economic burden on society [1, 2]. Currently, clinical treatments for refractory low back pain are limited, and there is an urgent need to improve the diagnosis and treatment of this condition [2]. Ninety percent of low back pain cases are nonspecific, and the intervertebral discs are an important source of low back pain [3, 4]. Discogenic low back pain is the most common type of chronic low back pain, accounting for 39% of cases [5].

The main pathophysiologic process of discogenic low back pain is the inflammatory response, in which inflammatory factors stimulate pain receptors, leading to mechanical nociceptive hypersensitivity [6, 7, 8]. Evidence suggests that degenerated cartilage endplates (CEPs), which have nerves and blood vessels growing into them, are the main source of discogenic low back pain [9, 10]. Abnormal mechanical stress is the main driving force in the development of discogenic low back pain [11]. It is currently thought that CEP degeneration may be the initiating factor for disc degeneration and hence low back pain [12, 13]. Piezo2 is a mechanical signaling channel that plays a key role in a variety of pathophysiological processes [14, 15, 16]. It mediates the sensitization of sensory nerves to mechanical stimuli in inflammatory environments [17, 18, 19]. Dorsal root ganglion (DRG) Piezo2 positivity in nerves innervating the CEP accounts for more than 90% of discogenic low back pain cases [10]. Therefore, we hypothesized that high concentrations of inflammatory factors in the degenerating CEP lead to hyperactivation of Piezo2 in the DRG, which sensitizes the organism to innocuous mechanical stimuli and in turn leads to discogenic low back pain.

In addition to transmitting pain, nerves are involved in the direct regulation of functions related to the cells they innervate. In the intervertebral disc, it has been proposed that sensory nerves maintain extracellular matrix homeostasis in nucleus pulposus cells through the CGRP/CHSY1 axis [20]. In contrast, interaction of the CEP with nerves, as a conduit for the exchange of nutrients and metabolic waste in the intervertebral disc [21], has rarely been reported. In osteoarthritis, in addition to pain transmission, the direct regulation of chondrocytes by neuropeptides, including chondrocyte extracellular matrix remodeling, apoptosis, proliferation, and inflammatory responses, has increasingly attracted attention from researchers in recent years [19, 22]. The CEP, although similar to articular cartilage, differs in terms of its developmental origin, tissue arrangement, and extracellular matrix composition and should be studied as a separate tissue structure [21]. In discogenic low back pain, interactions between sensory nerves and the CEP are likely involved in the process of intervertebral disc degeneration. Identifying the key molecular mechanisms involved in this process may provide promising clinical targets for the treatment of low back pain and intervertebral disc degeneration.

In the present study, we established a model of discogenic low back pain caused by lumbar spine instability surgery and explored the signaling channels that play key roles in discogenic low back pain and their interactions with the CEP. The results showed that Piezo2 in DRG neurons mediated the mechanical pain response in our rat model of discogenic low back pain and promoted activation of the NF‐κB pathway, the release of inflammatory factors in CEP cells, and the sensitization of neuronal mechanoreception through the CGRP/RAMP1/IKKβ axis, forming a positive feedback loop that further exacerbated intervertebral disc degeneration and low back pain. In terms of potential treatments, we used direct knockdown of Piezo2 expression and a dietary supplementation with ω‐3 polyunsaturated fatty acid eicosapentaenoic acid (EPA) strategy targeting Piezo2, as well as an oral small molecule antagonist, Rimegepant, which targets CGRP. On the basis of the above mechanisms, both of these strategies mitigated pain‐related behaviors and maintained the homeostasis of CEP cells.

## 2 Results

### 2.1 The CEP is the Site of the Most Pronounced Stress‐Related Changes in Models of Lumbar Spinal Instability

**FIGURE 1:**
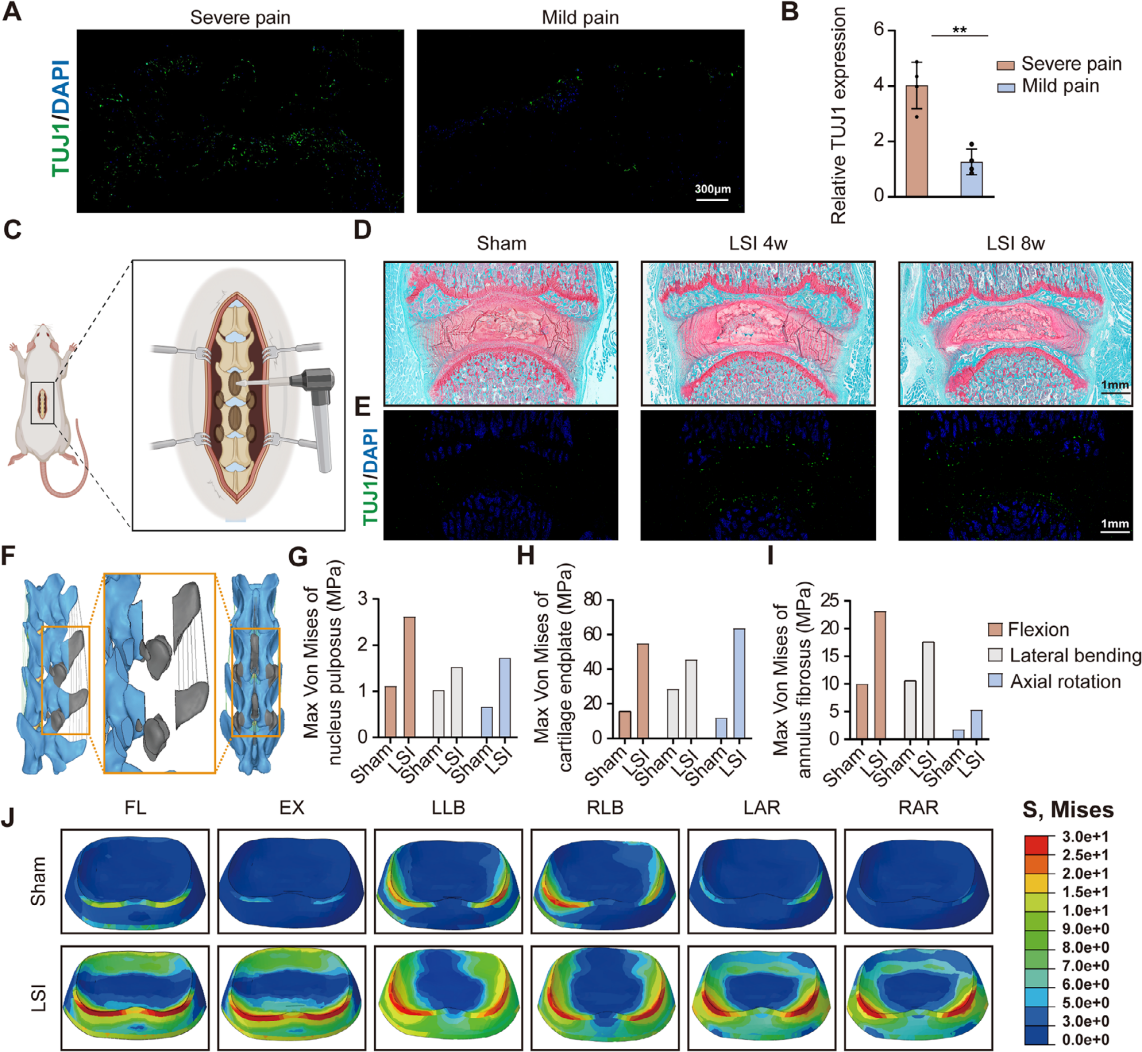
The CEP Exhibits the Most Pronounced Mechanical Stress Alterations with Concomitant Sensory Nerve Ingrowth in the LSI Model. Representative immunofluorescence images of TUJ1‐positive nerves (green) in CEP samples from patients with (A) severe and mild low back pain, with (B) a quantitative analysis of nerve density. (C) Schematic of the LSI surgery. (D) Coronal SO/FG‐stained sections of intervertebral discs from LSI versus sham‐operated rats, showing CEP degeneration. (E) Increased abundance of TUJ1‐positive nerve fibers (orange) in the caudal CEP of LSI rats. (F) Finite element model of the microstructure of the rat spine, with gray areas indicating LSI resection sites. Maximum von Mises stress values during motion with six degrees of freedom in the (G) nucleus pulposus, (H) CEP, and (I) annulus fibrosus of L4‐5 discs from the LSI and sham groups, alongside (J) stress distribution maps. Statistical significance was determined by Student's *t*‐test (**p < 0.01).

In the lumbar spine instability (LSI) model, we systematically evaluated the responses of various disc structures to mechanical stress alterations via finite element analysis and histologic methods. Immunofluorescence staining of CEP samples from patients with severe versus mild low back pain revealed that the infiltration density of TUJ1‐positive nerve fibers was significantly greater in the CEP of the severe pain group (Figure 1A,B), suggesting that there is a correlation between abnormal nerve growth and the severity of pain. To further clarify the mechanical microenvironmental alterations in the CEP, we constructed a rat LSI model (Figure 1C), in which coronal tissue staining revealed degeneration of the CEPs of the intervertebral discs in the surgery group (Figure 1D). Notably, immunofluorescence staining revealed a significant increase in nerve distribution in the caudal CEP region in the LSI group (Figure 1E), which was consistent with clinical observations.

By building a finite element model based on the 3D anatomical structure of the rat spine (Figure 1F), we quantitatively analyzed the stress distribution characteristics of the disc components during motion with six degrees of freedom. The maximum von Mises stress value of the CEP in the L4‐5 segment of the LSI group was significantly greater than that of the sham‐operated group, and the increase in CEP stress (Figure 1G–I) was significantly greater than that in the nucleus pulposus and the annulus fibrosus, with peak values as much as six times greater. The stress distribution map further revealed that the CEP was subjected to high stress concentrations during motion (Figure 1J). These results suggest that mechanical overload of the CEP, which is a core target of mechanical stress transmission, may initiate a pain signaling cascade by activating mechanosensitive channels in nerve endings.

### 2.2 Lumbar Spine Instability Leads to Low Back Pain‐Related Behavior and CEP Degeneration

**FIGURE 2:**
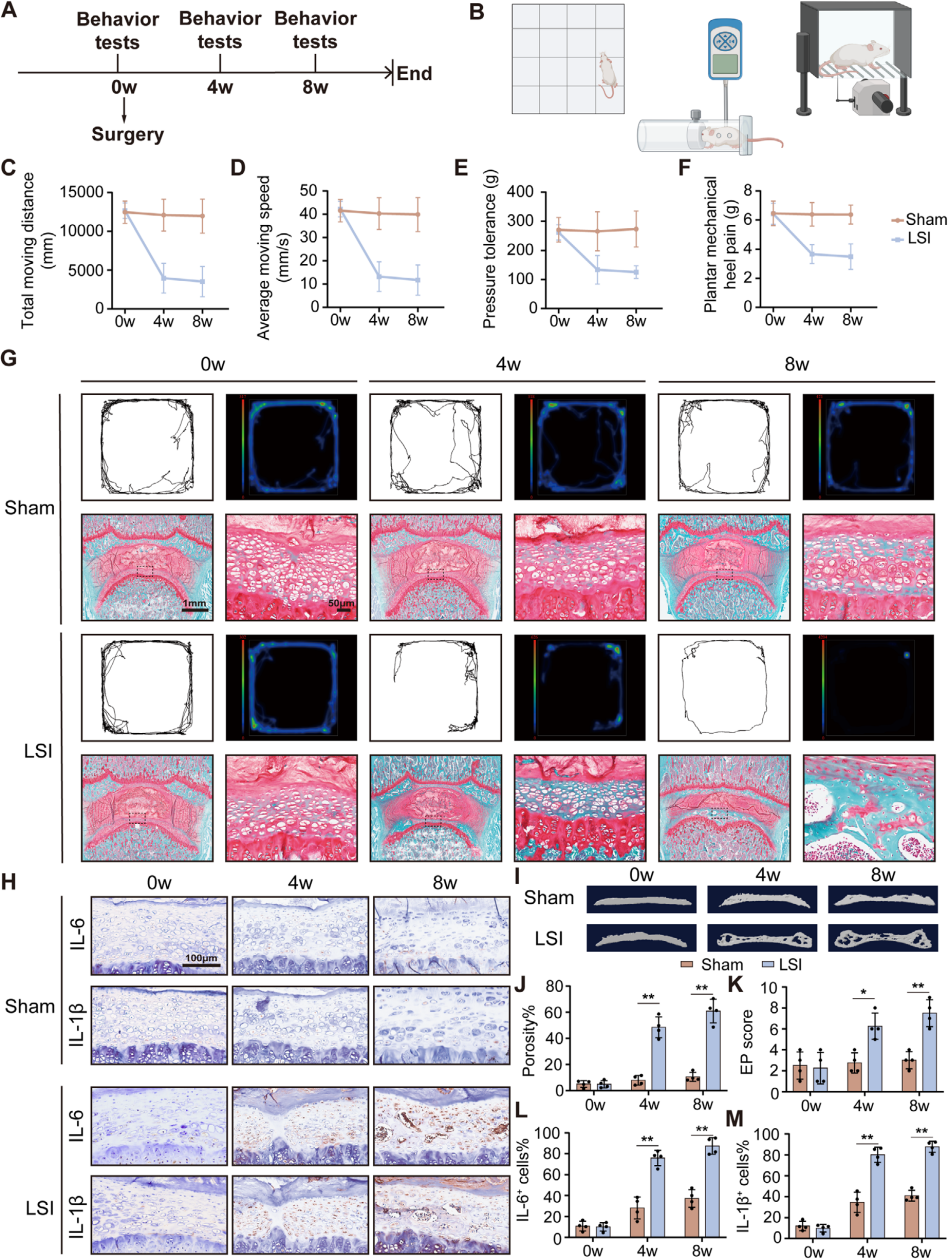
LSI Induces Low Back Pain‐Related Behaviors in Rats with Concurrent CEP Degeneration and Inflammatory Cytokine Upregulation (A) Schematic diagram of the LSI surgery and behavioral testing protocols. (B) Illustrations of the open field, dorsal mechanical pressure, and plantar nociception tests. Quantitative comparisons between LSI‐ and sham‐operated rats: (C) total travel distance and (D) average velocity in the open field test; (E) mechanical pressure tolerance over the L4–L5 dorsal region; and (F) plantar mechanical withdrawal thresholds (*n* = 5). (G) Upper panel: Open field movement trajectories at three postoperative timepoints; lower panel: corresponding representative SO/FG‐stained disc sections. (H) Representative immunohistochemical (IHC) staining of caudal L4‐L5 CEPs. (I) Coronal 3D micro‐CT reconstructions of caudal L4‐5 CEPs. (J) Quantitative analysis of total porosity. (K) Caudal CEP degeneration scores based on panel G. (L,M) IHC quantification of IL‐6 and IL‐1β in CEPs. Statistical significance was determined by Student's *t*‐test (*p < 0.05, **p < 0.01).

After establishing a rat model of LSI, we systematically assessed the temporal effects of abnormal mechanical stress on pain‐related behavior and CEP degeneration. Behavioral analyses revealed that the rats in the LSI group presented typical pain‐related behavioral features beginning in the fourth week post‐surgery: the total distance traveled (Figure 2C) and average speed (Figure 2D) of movement in the open‐field experiments decreased compared with those in the sham‐operated group, suggesting that the ability to perform voluntary movements was limited by pain; at the same time, there was a decrease in the pressure tolerance threshold for mechanical stimulation (MS) in the L4‐L5 back region (Figure 2E) and a decrease in the plantar tingling threshold (Figure 2F). Notably, pain‐related behavior peaked at 8 weeks post‐surgery, and the open‐field movement trajectory revealed significantly inhibited spatial exploration (Figure 2G), which was consistent with CEP degeneration of the intervertebral disc during the same period (lower panel of Figure 2G).

Histopathological analysis further revealed the spatiotemporal correlation between CEP degeneration and pain‐related behavior. Micro‐CT 3D reconstruction revealed that endplate porosity increased in the LSI group compared with the control group as early as week 4 (Figure 2I,J) and progressively worsened over time. Immunohistochemistry revealed an increase in inflammatory factors near CEP at 4 weeks post‐surgery (Figure 2H) and an elevated endplate degeneration score compared with that of the control group (Figure 2K). There were increases in the expression levels of IL‐6 and IL‐1β in the degenerating CEP (Figure 2L,M), and the degree of inflammatory factor accumulation was positively correlated with the severity of pain‐related behaviors during the same period. This cascade of endplate microinjury, inflammatory microenvironment development, and exacerbation of pain‐related behaviors triggered by abnormal mechanical loading suggests that CEP degeneration is not only a structural alteration resulting from mechanical stress imbalance but also an important pathological mechanism driving the continuous worsening of pain.

### 2.3 Key Role of the Piezo2/CGRP Axis in the Mechanical Stress and Nociceptive Transduction Pathway

**FIGURE 3:**
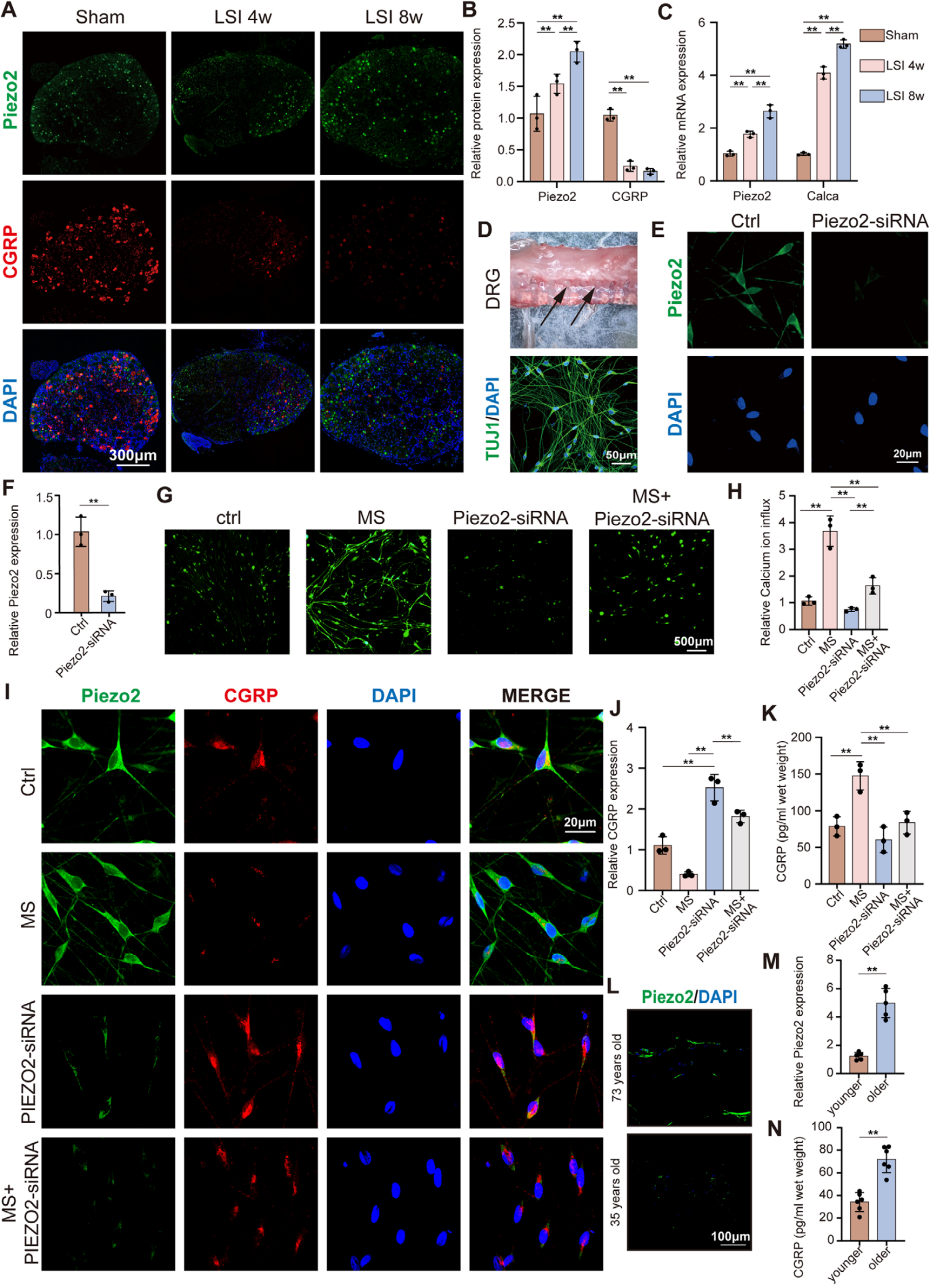
Piezo2 in Neurons Senses Mechanical Stimuli to Drive CGRP Production via Calcium Influx (A) Representative immunofluorescence images of Piezo2 (green) and CGRP (red) in L4‒5 dorsal root ganglia (DRGs) from lumbar spinal instability (LSI) and sham‐operated rats, with (B) quantification of Piezo2‐positive neurons and intracellular CGRP levels. (C) The relative expression levels of mRNAs encoding Piezo2 and CGRP in DRG tissues (D) Upper panel: Schematic diagram of the DRG anatomical location; lower panel: TUJ1 (green) immunofluorescence confirming neuronal identity. (E) Piezo2 immunofluorescence and (F) quantification in DRG neurons after siRNA‐mediated Piezo2 knockdown. (G) Representative fluorescence images of calcium influx and (H) quantification for the control (Ctrl), mechanical stimulation (MS), Piezo2‐siRNA, and MS+Piezo2‐siRNA groups. (I) Piezo2 (green) and CGRP (red) immunofluorescence in the experimental groups. (J) Intracellular CGRP expression and (K) CGRP concentration in supernatants. (L) Representative images of Piezo2 immunofluorescence staining in clinical cartilage endplate specimens from young and elderly patients, and (M) quantitative analysis. (N) Elisa analysis results of clinical cartilage endplate specimens. Statistical significance was determined by one‐way ANOVA and Student's *t*‐test (**p < 0.01).

Previous studies have demonstrated the involvement of Piezo2 in the process of nociceptive sensitization as a core mechanotransduction channel, but its specific mechanism of action in discogenic low back pain has not been elucidated. By integrating neurobiological and biomechanical analyses, we revealed a novel mechanism by which abnormal mechanical stress drives pain signal amplification through the Piezo2/Ca^2^^+^/CGRP axis. Immunofluorescence quantification showed a significant increase in the proportion of Piezo2‐positive neurons in L4‐L5 segment DRGs of the LSI group at 4‐ and 8‐weeks post‐surgery (Figure 3A,B), whereas intracellular CGRP expression was reduced. Meanwhile, qPCR analysis demonstrated sustained upregulation of Calca in DRG tissues at these two time points (Figure 3C). This seemingly paradoxical phenomenon suggests that mechanical stress (MS) may reduce intracellular CGRP storage by promoting its secretion. This hypothesis was verified in a primary DRG cell model: intracellular CGRP levels decreased in the MS group compared with those in the control group, but the CGRP concentration in the supernatant increased (Figure 3J,K), suggesting that abnormal stress participates in pain signaling by triggering the rapid secretion of CGRP. To investigate whether other pathways, such as PKC/cAMP signaling, extracellular ATP, and central sensitization, were involved, we performed PCR experiments to assess the mRNA expression levels of key molecules in DRG tissue (Figure S1). The results showed that, compared with the sham‐operated control group, these genes were not significantly upregulated in LSI rats.

Piezo2 is localized to the plasma membrane (Figure S2). To clarify the central role of Piezo2 in this process, we extracted primary DRG cells (Figure 3D) and knocked down Piezo2 expression via siRNA (Figure 3E,F). Calcium imaging experiments revealed that MS enhanced Ca^2^^+^ influx in control DRG cells, whereas Piezo2 knockdown largely blocked the calcium response (Figure 3G,H), confirming that Piezo2 is a key mediator of mechanical‒chemical signaling. Notably, DRG cells in the MS group presented a decrease in intracellular CGRP fluorescence intensity compared with those in the control group, in parallel with high Piezo2 expression, but this phenomenon disappeared in the Piezo2‐knockdown group, suggesting that mechanical stress promotes CGRP secretion (Figure 3I–K). In contrast, clinical specimens from older patients showed higher expression of Piezo2 in cartilage endplate (Figure 3L,M), and Elisa analysis revealed elevated CGRP levels within their tissues (Figure 3N).

### 2.4 Superimposition of Abnormal Stress and Neuropeptide Phases Mediates CEP Degeneration

**FIGURE 4:**
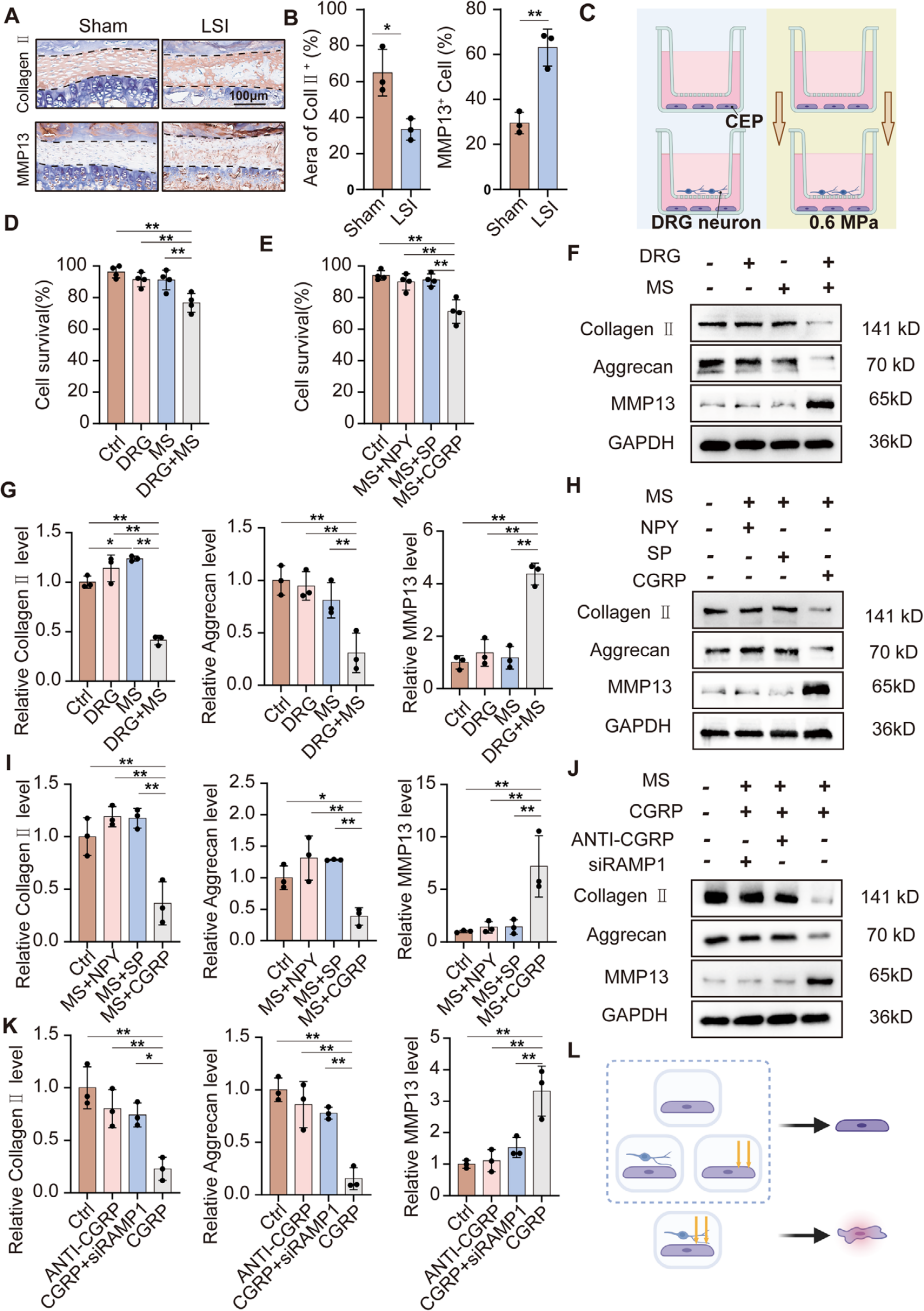
Abnormal Stress‐Mediated CEP Degeneration is CGRP/RAMP1 Dependent (A) Representative IHC staining and (B) quantification of Collagen II and MMP13 expression in CEP tissues from the sham‐operated and lumbar spinal instability (LSI) groups. (C) Schematic diagram of the coculture system for DRG neurons and CEP cells. (D) Cell viability assay in CEP cells under four conditions: control (ctrl), DRG coculture (DRG), mechanical stimulation (MS), and DRG+MS. (E) Cell viability assay in CEP cells subjected to MS with or without NPY, SP, or CGRP. (F, G) Western blot analysis of Collagen II, Aggrecan, and MMP13 expression in CEP cells under four conditions: control (ctrl), DRG coculture (DRG), mechanical stimulation (MS), and DRG+MS. (H,I) Western blot analysis of Collagen II, Aggrecan, and MMP13 expression in CEP cells subjected to MS with or without NPY, SP, or CGRP. (J, K) Western blot analysis of Collagen II, Aggrecan, and MMP13 expression in CEP cells under MS with or without CGRP, an anti‐CGRP antibody, or RAMP1 silencing. (L) Schematic diagram: CEP degeneration occurs only under combined MS and DRG coculture. Statistical significance was determined by one‐way ANOVA and Student's *t*‐test (*p < 0.05, **p < 0.01).

On the basis of these findings, we further explored the critical role of CGRP in CEP degeneration. Compared with that in the sham‐operated group, Collagen II expression in the CEP in the LSI group was decreased, whereas MMP13 expression was increased (Figure 4A,B), suggesting severe dysregulation of the synthesis‒catabolism balance of the extracellular matrix. To clarify whether this phenomenon was triggered by synergistic mechanical–neural signaling, we established a Transwell coculture system with DRG neurons and CEP cells (Figure 4C) and found that only when MS (0.6 MPa) and DRG cocultures were applied simultaneously was CEP cell viability decreased (Figure 4D) and Collagen II and aggrecan expression reduced. Moreover, the MMP13 level was elevated (Figure 4F,G). This dual signaling dependence was further validated via a neurotransmitter screen: the addition of CGRP alone (but not NPY or SP) exacerbated the degradation of the CEP extracellular matrix in response to MS (Figure 4E,H,I), and this effect was completely blocked by CGRP‐neutralizing antibodies or RAMP1 gene silencing (Figure 4J,K).

Notably, the application of 0.6 MPa mechanical stress alone (corresponding to the abnormal stress level of the CEP in the LSI model) did not result in significant matrix metabolic imbalance (Figure 4L). These findings suggest that CEP degeneration requires the synergistic activation of abnormal stress with CGRP signaling.

### 2.5 CGRP Activates the NF‐κB Axis in CEP Cells by Upregulating IKKβ Expression in Response to Abnormal Stress Stimulation

**FIGURE 5:**
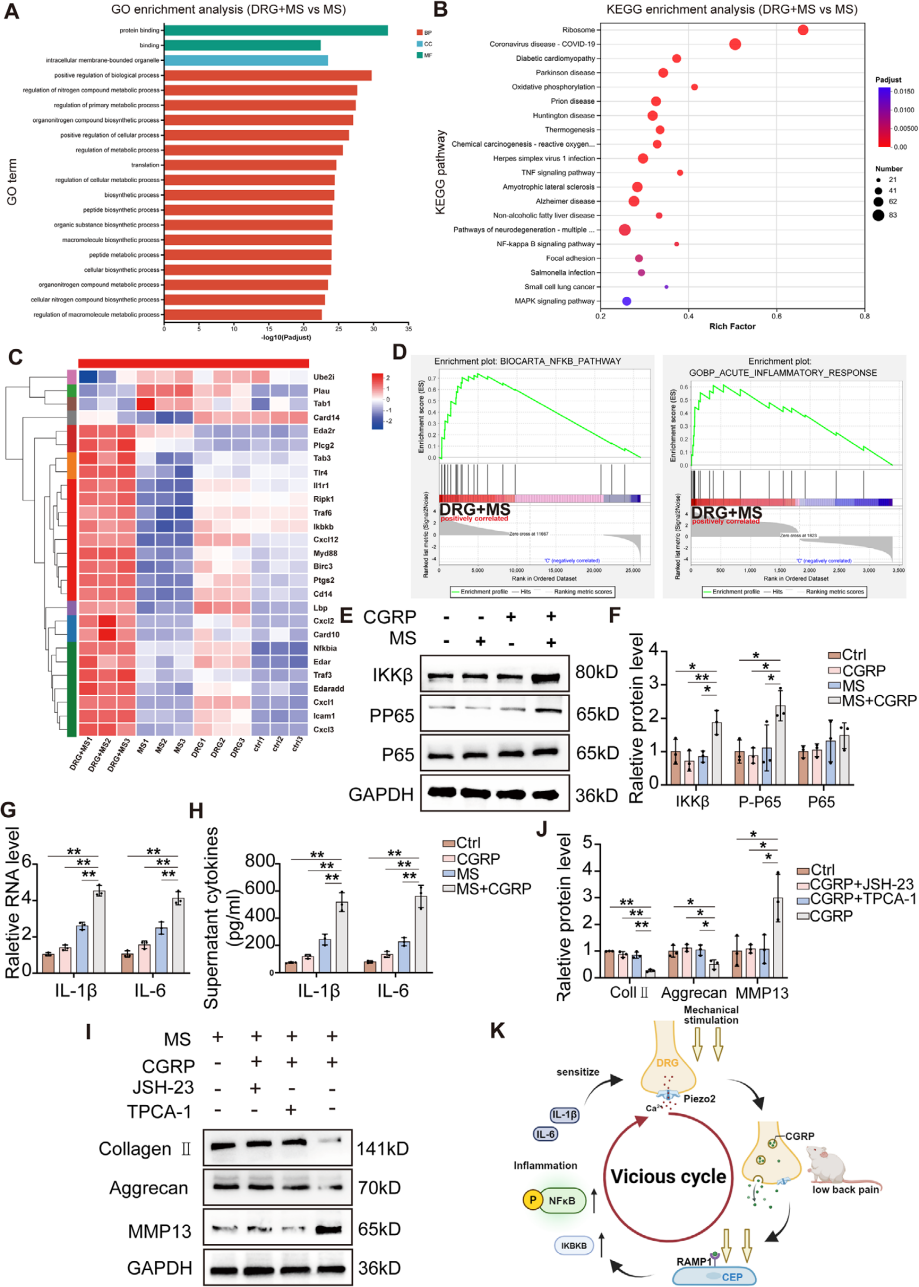
CGRP upregulates IKKβ expression to activate the NF‐κB axis under mechanical stimulation. (A) Gene Ontology (GO) and (B) Kyoto Encyclopedia of Genes and Genomes (KEGG) pathway enrichment analyses of differentially expressed genes in CEP cells cultured with or without DRG cells under MS. (C) Heatmap of NF‐κB pathway‐related genes expressed in CEP cells cultured with or without DRG cells and with or without MS. (D) Gene set enrichment analysis (GSEA) of CEP cells cultured with or without DRG cells under MS. (E,F) Western blot analysis of IKKβ, P65, and P‐P65 expression in CEP cells with or without the addition of CGRP and with or without MS. (G) PCR analysis of IL‐1β and IL‐6 mRNA expression. (H) Determination of the IL‐1β and IL‐6 levels in the cell supernatants. (I, J) Western blot analysis of Collagen II, Aggrecan, and MMP13 expression in CEP cells subjected to MS with or without CGRP, JSH‐23, or TPCA‐1. (K) Schematic diagram: DRG neurons sense MS, generating low back pain and releasing CGRP. CGRP activates the NF‐κB pathway in CEP cells under MS, leading to the secretion of inflammatory factors, which sensitize neurons, creating a positive feedback loop. Statistical significance was determined by Student's *t*‐test (*p < 0.05, **p < 0.01).

On the basis of the dual signaling dependence of CEP degeneration in the coculture system, we performed transcriptome sequencing of four groups of CEPs with and without neural cell coculture or MS, and the results revealed that the genes differentially expressed between CEP cells in the mechanically stimulated group and those in the DRG coculture group were significantly enriched in the inflammatory response and NF‐κB signaling pathways (Figure 5A,B). Heatmap analysis further confirmed that the expression of a series of key genes in the NF‐κB pathway was upregulated only in the presence of both MS and DRG coculture (Figure 5C), and gene set enrichment analysis (GSEA) revealed that the set of NF‐κB target genes and inflammatory response genes was significantly activated (Figure 5D).

At the molecular level, CGRP synergized with mechanical stress to activate the NF‐κB pathway by upregulating IKKβ (IKBKB). Western blot analysis revealed that combined MS and CGRP treatment significantly increased IKKβ protein levels and phosphorylated p65 (p‐p65) levels in CEP cells (Figure 5E,F), whereas either stimulus alone had minimal effects. Consistent with these findings, the results of the qPCR assays revealed increased levels of IL‐1β and IL‐6 mRNAs (Figure 5G) and increased concentrations of the corresponding proteins in the cell supernatants (Figure 5H). When the NF‐κB inhibitors JSH‐23 (which blocks nuclear translocation) and TPCA‐1 (which inhibits IKKβ kinase activity) were used, Collagen II and Aggrecan expression could be restored to the control level even in the presence of MS and CGRP (Figure 5I,J), confirming that the IKKβ/NF‐κB axis is a key hub for the synergistic effect of CGRP and mechanical forces.

The above regulatory network reveals a key positive feedback loop in the progression of discogenic low back pain: abnormal mechanical stress in the CEP activates DRG neurons through Piezo2, which leads to the release of CGRP; CGRP binds to the RAMP1 receptor in CEP cells under the synergistic effect of mechanical stress, which upregulates the expression of IKKβ, activates the NF‐κB pathway, and drives the secretion of inflammatory factors such as IL‐1β/IL‐6 (Figure 5K). Previous studies have shown that inflammatory factors can promote the sensitivity of Piezo2 in dorsal root ganglion (DRG) neurons to mechanical stimuli, thereby establishing a positive feedback loop mechanism in which inflammation induces neural sensitization and neural sensitization exacerbate inflammation.

**FIGURE 6:**
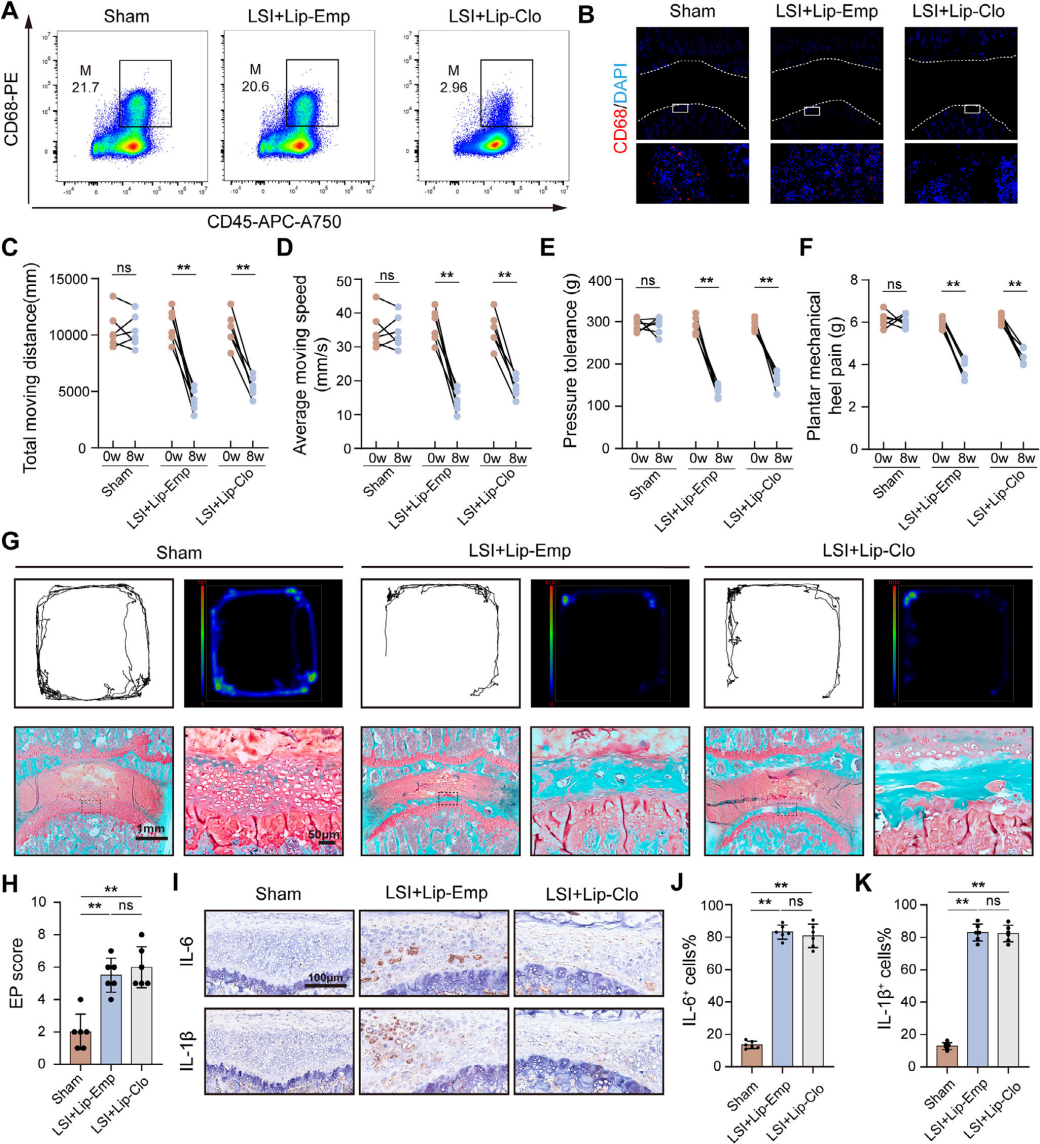
After macrophage depletion, LSI rats still exhibited significant low back pain‐related behaviors and the accumulation of inflammatory factors near the cartilaginous endplate. (A) Flow cytometric analysis of CD68+CD45+ macrophage cells in the spleen of sham surgery group (sham), post‐LSI tail vein injection of blank liposomes group (LSI+Emp), and Clodronate Liposomes group (LSI+Clo). (B) Representative immunofluorescence images of macrophages near the cartilaginous endplates of intervertebral discs in three groups of rats. Quantitative comparisons of behavioral experiments among the three groups of rats: (C) total travel distance and (D) average velocity in the open field test; (E) mechanical pressure tolerance over the L4–L5 dorsal region; and (F) plantar mechanical withdrawal thresholds (*n* = 6). (G) Upper panel: Open field movement trajectories; lower panel: corresponding representative SO/FG‐stained disc sections. (H) Caudal CEP degeneration scores based on panel G. (I) Representative immunohistochemical (IHC) staining of caudal L4‐L5 CEPs. (J,K) IHC quantification of IL‐6 and IL‐1β in CEPs. Statistical significance was determined by Student's *t*‐test (*p < 0.05, **p < 0.01).

However, the immune system participates in inflammatory responses by releasing relevant inflammatory mediators. To rule out interference from the immune system in the aforementioned process, we conducted additional animal experiments. Under normal conditions, the intervertebral disc is regarded as an immune‐privileged organ. During disc degeneration, a small number of immune cells may infiltrate the disc tissue, particularly in the vicinity of the cartilaginous endplates, with macrophages accounting for the overwhelming majority of these cells. Therefore, we induced systemic macrophage depletion in rats via twice‐weekly tail vein injections of Clodronate Liposomes. After 8 weeks, flow cytometric analysis of spleen tissue demonstrated a significant reduction in macrophages in Clodronate Liposomes ‐treated rats (Figure 6A). Additionally, immunofluorescence staining revealed a marked decrease in macrophage infiltration near the cartilage endplates (Figure 6B). Behavioral experiments showed that following macrophage depletion, LSI rats still exhibited significantly increased lumbar pain‐related behaviors compared to their preoperative levels, accompanied by degenerative changes in the cartilage endplates (Figure 6C–H). Immunohistochemical analysis indicated no significant reduction in IL‐6 and IL‐1β levels within the cartilage endplates, suggesting that these cytokines are not primarily secreted by macrophages in the LSI model (Figure 6I–K). Collectively, these findings indicate that in vivo, mechanical homeostasis imbalance triggers degenerative changes in the cartilage endplates, which in turn promote the production of large quantities of inflammatory mediators, thereby perpetuating a vicious cycle.

### 2.6 Targeting Piezo2 and CGRP Reduces Pain‐Related Responses and Delays Progression of Degeneration

**FIGURE 7:**
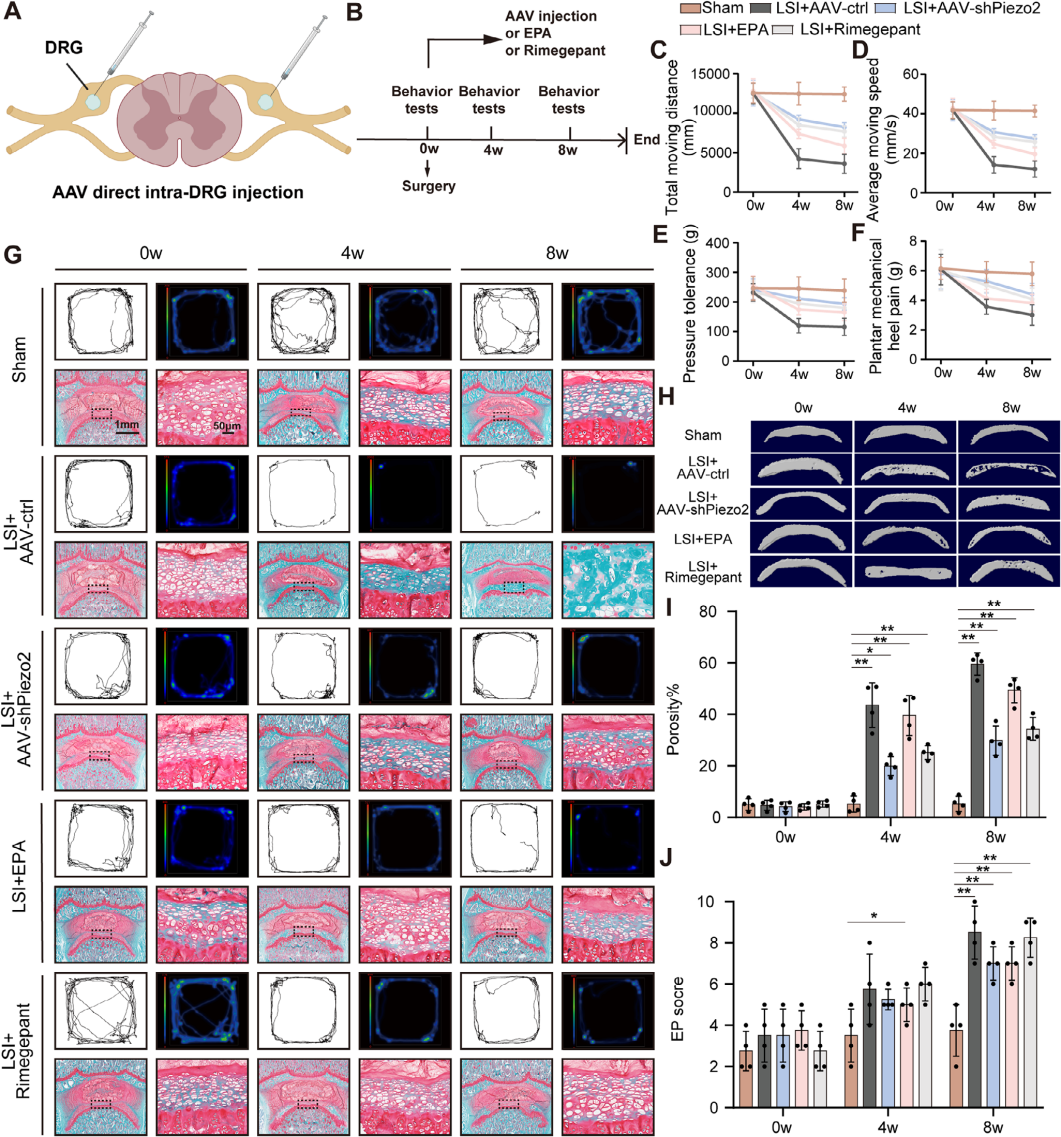
Targeting Piezo2 and CGRP reduces pain‐related responses and slows the progression of degeneration. (A) Schematic diagram of direct injection of AAV‐shPiezo2 into the rat DRG to knock down Piezo2. (B) Schematic diagram of the administration of a EPA dietary supplement or oral Rimegepant to rats after LSI. (C) Distance moved in the open field and (D) average speed. (E) Mechanical pressure tolerance above L4‐L5 and (F) paw withdrawal threshold, *n* = 5. (G) Upper: Open‐field trajectories of the five groups at three time points; Lower: Representative images after SOFG staining images. (H) Representative 3D micro‐CT images (coronal view) of L4‐5 caudal endplates. (I) Caudal cartilaginous endplate scoring based on (D). (J) Quantitative analysis of total endplate porosity. Statistical significance was determined by one‐way ANOVA (* *P* < 0.05, ** *P* < 0.01).

To validate the therapeutic potential of the Piezo2/CGRP signaling axis, two types of intervention regimens were designed in this study: (1) direct knockdown of Piezo2 expression in DRG neurons with AAV‐shPiezo2 (Figure 7A; Figure S3A,B) and dietary supplementation with EPA (300 mg/(kg∙d)) to inhibit Piezo2 channel activity; and (2) oral administration of the CGRP receptor antagonist Rimegepant. Micro‐CT revealed that at 8 weeks after LSI, endplate porosity was lower in the AAV‐shPiezo2 group and the Rimegepant group, respectively, than in the control group (Figure 7H), and the endplate degeneration scores were restored to those of the sham‐operated group (Figure 7J). Histological staining further confirmed that CEP degeneration was lower in the intervention group (Figure 7G, lower panel), suggesting that targeted therapy could effectively slow the endplate degeneration process.

Behavioral analyses revealed that both intervention strategies significantly alleviated pain‐related phenotypes. Compared with those in the control group, the rats in the AAV‐shPiezo2 group presented increased movement distance and speed (Figure 7C,D), and the recovery of dorsal mechanical pain thresholds and plantar nociceptive responses in the AAV‐shPiezo2 group was close to that in the sham‐operated group (Figure 7E,F). Notably, the degree of pain relief was greater in the Rimegepant group. Mechanistically, the expression of IL‐6 and IL‐1β in the CEP was lower in the intervention group than in the control group (Figure S4), suggesting that inhibition of the Piezo2/CGRP axis could exert a therapeutic effect by blocking the NF‐κB inflammatory loop.

Given that many patients only seek medical care after the onset of low back pain, we specifically simulated this clinical scenario in an animal model (Figure S5A). At 4 weeks post‐LSI surgery, rats displayed heightened low back pain‐related behaviors. At this time point, each rat was supplemented with additional EPA on a daily basis, followed by an 8‐week continuous observation period. Behavioral test results demonstrated that this EPA supplementation did not significantly alleviate low back pain‐related behaviors in the rats (Figure S5B–G). Histological examinations further revealed no significant improvement in cartilage endplate degeneration (Figure S5H–J). These findings suggest that EPA, when used as a dietary supplement, should be administered prophylactically prior to the initiation of the “mechanical homeostasis imbalance‐inflammation” vicious cycle.

## 3 Discussion

In this study, we systematically revealed the regulatory network of mechanical–neuroinflammatory interactions involved in discogenic low back pain by integrating biomechanical and molecular biological approaches. We found that abnormal mechanical stress on the CEP triggered CGRP release through the activation of Piezo2 channels in the DRG, whereas CGRP drove the secretion of inflammatory factors through the RAMP1/IKKβ/NF‐κB axis in concert with mechanical forces, creating a positive feedback loop.

While previous studies have focused mostly on the direct mechanical stresses that lead to structural damage to the disc [23, 24], the present study revealed that CEP degeneration requires the synergistic action of mechanical forces and neural signals. Although a mechanical stress of 0.6 MPa alone was not sufficient to disrupt CEP homeostasis, it could induce CGRP secretion through the activation of Piezo2, which in turn promoted the degenerative process by enhancing the inflammatory response of CEP cells to mechanical stress. The present study clarified for the first time the specificity of Piezo2 as a hub for mechanical‒chemical signaling interactions and revealed the dual‐factor dependent activation of the NF‐κB pathway, which is characteristic of this process. This finding suggests that the key to treating discogenic low back pain lies in blocking mechanical signaling to disrupt this positive feedback loop. Although our data highlight the role of the Piezo2/CGRP axis as a key driver, other pathways (such as PKC/cAMP signaling) may be involved in the maintenance of chronic pain, particularly in later stages, and warrant further investigation in the future. It should be noted that Piezo1 is also expressed in sensory neurons and may partially compensate for the physiological functions of Piezo2 under specific conditions. The regulatory roles of each can be further elucidated using a double‐knockout model in future studies.

Since there are no specific small molecule inhibitors for Piezo2 and systemic interventions can affect normal physiological functions, there are no reliable clinical treatments targeting Piezo2 [25, 26]. Some studies have shown that different types of fatty acids can modulate the mechanoreceptive ability of Piezo2 [27]. For example, a linoleic acid‐enriched diet increases Piezo2 activity, in Angelman syndrome mice [28]. Moreover, neuro‐electrophysiological experiments have shown that dietary fatty acid margaric acid decreases Piezo2 function [29]. Additionally, dietary supplementation with EPA was recently shown to alleviate distal arthrogryposis caused by Piezo2 dysfunction [16]. Similarly, dietary supplementation with EPA before LSI in our study resulted in some degree of low back pain relief, with degeneration‐delaying effects (Figure 7F). EPA is commonly found in fish and widely used as a dietary supplement. When people engage in work that places significant strain on the lower back, supplementing with EPA appears to be a good option for preventing low back pain.

In recent years, the neuropeptide CGRP has been found to play key roles in various physiological processes [30, 31]. Interestingly, this neuropeptide has opposite effects on health in different scenarios. In the intervertebral disc, CGRP promotes homeostasis of the extracellular matrix of nucleus pulposus cells [20]. However, the studies reporting this effect did not focus on the effects of mechanical stimuli. Intervertebral disc degeneration occurs in a generalized environment of stress imbalance, and CGRP has negative effects in the presence of mechanical stimulation. We believe this phenomenon reconciles seemingly contradictory observations in this field: CGRP exerts a protective effect on the NP when biomechanical stability is preserved, whereas it accelerates CEP damage when mechanical homeostasis fails. In the present study, the CGRP receptor antagonist Rimegepant slowed CEP degeneration by blocking the inflammatory cascade through the inhibition of CGRP signaling. These findings suggest that focusing on the role of CGRP under different conditions could reveal new ideas for treating diseases. Furthermore, following nerve injury, glial cells and Schwann cells interact closely with sensory neurons. Whether Piezo2 in non‐neuronal cells is involved in regulating mechanical hyperalgesia and maintaining chronic pain requires further validation using cell‐specific conditional knockout models.

Although this study demonstrated the pivotal role of the Piezo2/CGRP axis in low back pain, the following limitations remain: (1) the rat LSI model could not fully mimic the chronic and progressive nature of human discogenic low back pain; (2) the intervention window (immediate postoperative dosing) may not be consistent with the chronic stage of discogenic low back pain in patients; and (3) the effect of long‐term treatment on the overall biomechanical properties of the disc was not evaluated. Although adeno‐associated virus‐mediated interference of Piezo2 in dorsal root ganglia effectively alleviates pain‐related behaviors, the regulatory role of Piezo2 in spinal interneurons cannot be ruled out. Further validation will require the development of conditional knock‐out models for Piezo2 in dorsal root ganglia and spinal neurons, respectively. At the same time, it should be clarified which neuronal subpopulations play the primary role.

In summary, the present findings reveal an important mechanism underlying the progression of discogenic low back pain and provide a theoretical basis for the development of a multitargeted mechanical–neurological therapeutic strategy.

## 4 Materials and Methods

### 4.1 Clinical Specimens

All CEP samples were obtained from surgical patients, and the study was conducted after patients provided informed consent. Ethical approval was obtained from the Ethics Committee of Tongji Medical College, Huazhong University of Science and Technology (NO. 0448).

### 4.2 Finite Element Analysis

Micro‐CT images of the lumbar spine of normal 8‐week‐old male rats were used in this study, and solid models were subsequently constructed and meshed with Mimics (Materialise, Belgium) and HyperMesh software (Altair Engineering, Inc., USA). Finite element simulation analysis was performed using Abaqus (Dassault, France) software. The model included the vertebral body, intervertebral discs, endplates, and ligaments. Each part of the model was assigned the appropriate material properties as described in the literature [10, 24].

### 4.3 Animal Experiments

#### 4.3.1 Lumbar Instability Model

Sprague‐Dawley rats were purchased from the Laboratory Animal Center of Huazhong University of Science and Technology (Wuhan, China). The surgical procedure was approved by the Animal Experimentation Committee of Huazhong University of Science and Technology (NO. 4592). Male SD rats (8 weeks old; weighing approximately 200 g) were divided into the following groups: the sham‐operated, LSI, LSI+ AAV‐shPiezo2, LSI+ AAV‐Ctrl, LSI+EPA, and LSI+Rimegepant groups (at 0 w, 4 w, and 8 w, behavioral experiments were performed; samples were collected at 4 and 8 w; *n* = 5 per group). LSI model rats were anesthetized via an intraperitoneal injection of sodium pentobarbital (60–80 mg/kg). After the paravertebral muscles on both sides of the spinous process were cut, the L3–L5 supraspinous ligaments, the interspinous ligaments, and the posterior and posterolateral halves of the joints on both sides were resected sequentially. Sham‐operation group only cut the skin and separated the muscles dully. Penicillin (19 mg/0.1 mL) was injected intramuscularly immediately after surgery. After surgery, the rats were individually housed in plastic cages with a sawdust floor and supplied with water and food ad libitum [9, 32].

#### 4.3.2 DRG Injection In Situ

Animal surgery was performed as described previously, with a midline incision made over the spine at the L3‐L5 level and L5 identified at the midpoint of the line between the iliac crests on either side. A 0.8‐mm hole was drilled in the transverse process on the L4 and L5 DRGs (1 mm wide at the lower edge of the transverse process). The proximity of the ganglion was verified by paw twitching. A microsyringe containing 2 µL of shRNA‐AAV (GENERAL BIOL) mixture was inserted through the hole into the DRG to a depth of 500 µm. The shRNA‐AAV particles were slowly injected into the L4, and L5 DRGs on one side, and the needle was withdrawn 2 min after the injection was completed [33, 34]. The shRNA sequence targeting rat Piezo2 was available on request.

#### 4.3.3 Macrophage Depletion

Rats were injected with Clodronate Liposomes (YEASEN, Shanghai, China) via the tail vein at a dose of 10 µL/g twice a week for 8 weeks, while the control group was injected with blank Liposomes (PBS).

#### 4.3.4 Flow Cytometry

Prepare a single‐cell suspension of rat spleen. For each sample, 2,000,000 cells were added into a tube, blocked for 30 min at 4°C. Stained with 100 times diluted APC/Fire 750 anti‐rat CD45 (No.202221, BioLegend, USA), PE anti‐rat CD68 Recombinant Antibody (No.201003, BioLegend, USA). Flow cytometry was performed using DxFLEX Flow Cytometer (BECKMAN COULTER, USA) and data were recorded for the corresponding channel.

#### 4.3.5 Animal Behavioral Testing

A behavioral assessment was performed before surgery and 4,8 weeks after surgery. All behavioral assessments were performed by the same investigator, who was not known to the research team, to prevent assessment bias [10, 22, 32, 35].

##### 4.3.5.1 Pressure Hyperalgesia

Vocalization thresholds were measured on the basis of force applied from a dynamometer (Yaokun Biotechnology) by pressing the tip of a 5 mm diameter device directly onto the dorsal skin at L4‐L5 while gently restraining the rats. The force was increased at a rate of approximately 50 g/s until an audible grunt was heard. The cutoff force was 500 g to prevent tissue damage. Two tests were performed 15 min apart, and the average value was taken as the injurious threshold.

##### 4.3.5.2 Open Field (Assessment of Free Movement)

The rats were individually placed in a square observation box (75 × 75 cm) and allowed to explore freely for 5 min under normal lighting. The animal's movement trajectory was recorded by a computerized video tracking system, and parameters such as total movement distance and movement speed were analyzed.

##### 4.3.5.3 Electronic Von Frey Test

In each test, the rats were placed in a transparent plastic chamber (LWH 40 cm × 20 cm × 31 cm) suspended above a screened platform. After 15 min of habituation, the mechanical sensitivity of the right hind paw of each rat was examined using electronic von Frey filament (KEWBASIS, KW‐CT‐1). The filament was applied to the plantar surface five times with a 10‐s interval. For each trial, the filaments were applied until filament bending occurred and then held for 5 s. The retraction threshold was defined as the force value of the filament that produced three hindpaw retractions in response to five stimuli.

##### 4.3.5.4 Straight Leg Raise Test

The hind limb was extended (knee fully extended), and the hip was flexed for 2 s. The number of vocalizations in response to five leg extensions and lifts was recorded. Negative results in this test indicate that nerve root compression is not associated with the high nociceptive sensitivity that develops after LSI surgery [36, 37].

#### 4.3.6 Immunohistochemistry and Immunofluorescence

After removal, the samples were fixed for 24–48 h and decalcified at room temperature for 4 weeks. The samples were subsequently embedded in paraffin. Coronal paraffin sections 5 µm thick were generated from L4‒L5 segments of the lumbar vertebrae and subjected to Senna O‐Fixed Green (SOFG) staining and immunohistochemistry (IHC); 10 µm thick coronal frozen sections were used for immunofluorescence staining. The following antibodies were used: Collagen II (Affinity, AF0135); MMP13 (Proteintech, 18165‐1‐AP); IL‐1β (Proteintech, 26048‐1‐AP); IL‐6 (Proteintech, 21865‐1‐AP); CGRP (Abcam, ab81887); Piezo2 (Invitrogen, PA5‐72976); TUJ1 (Proteintech, 66375‐1‐Ig).

#### 4.3.7 Micro‐CT

The intact lumbar spine samples were dissected and scanned using a high‐resolution micro‐CT system (Skyscan1176, Bruker) with the following parameters: tube voltage of 65 kV, tube current of 385 mA, and aluminum filter thickness of 0.5 mm. The scanning data were reconstructed in three dimensions with the software accompanying the system (CT‐Vox; SkyScan CT). Coronal images of the L4‐L5 segments were selected for 3D histomorphometric analysis of the caudal endplate to assess the total porosity of the endplate. To visualize the 3D structural characteristics of the endplates, five consecutive layers of coronal images were selected for 3D modeling and visualization.

### 4.4 Cell Experiments

#### 4.4.1 Cell Culture

##### 4.4.1.1 DRG Cell Isolation

Suckling rats were sacrificed, and the trunk between the forelimbs and femur was isolated. The dorsal portions of the vertebrae and spinal cord were removed to facilitate the collection of the bilateral lumbar DRGs with microscissors. Neuronal fibers connecting the DRGs were removed to improve the purity of the cultures. DRG neurons were collected in cold neural basal medium and then treated with 3.5 mg/ml dispersase or 1.6 mg/ml type I collagenase dissolved in HBSS (Ca^2+^ and Mg^2+^ free) for 30 min at 37°C. After centrifugation, the cells were resuspended in neural basal medium supplemented with B27, 10% fetal bovine serum (Hyclone), and 1% penicillin‒streptomycin and spread in six‐well plates coated with poly L‐lysine (0.5 mg/ml) and laminin (10 lg/ml). The cells were incubated at 37°C in 5% CO2. DMEM/F12 complete medium was used for the first day of incubation, and Neurobasal (Procell, PM151223) + B27 (Procell, PM180637) complete medium was used beginning on the second day [31, 38].

##### 4.4.1.2 CEP Cell Isolation

CEP tissue was collected from the lumbar spine of 8‐week‐old rats. The tissues were digested in 2 mg/mL 0.1% type II collagenase (Gibco, 17101015) at 37°C for 4 h. The digested cartilage tissue was then incubated in DMEM supplemented with 10% fetal bovine serum (Hyclone) and antibiotics (1% penicillin/streptomycin), and the mixture was maintained at 5% CO2 and 37°C. After the cells reached confluence, they were harvested with 0.25% trypsin. The first three generations of CEP cells were used for the experiments [39, 40].

#### 4.4.2 Mechanical Stress Stimulation

Mechanical stress stimulation was applied to endplate chondrocytes and DRG neuronal cells using a cell compression culture system (Cell & Force Compressor). The cells were inoculated on the surface of the culture mold at an initial density of 1 × 10^4^ cells/cm^2^. The e parameters were set to 0.6 MPa for 24 h [41, 42].

#### 4.4.3 Real‐Time Fluorescent Quantitative PCR

CEPs in the Ctrl, DRG, MS, and DRG+MS groups were seeded in six‐well plates. After 48 h, total RNA was extracted using the FastPure Cell/Tissue Total RNA Isolation Kit V2. The RNA concentration and purity (A260/A280 ratio of 1.8–2.1) were measured with a microspectrophotometer. cDNA was synthesized from 1 µg of total RNA using HiScript III RT SuperMix (Vazyme, R323‐01). Real‐time PCR was performed with 2 µL of cDNA, 0.2 µM primers, SYBR (Vazyme, Q712‐02), and DEPC water in a 20 µL system. The program was 95°C for 30 s, followed by 40 cycles of 95°C for 5 s and 55°C for 10 s, with a melting curve analysis stage. GAPDH was used as the reference gene, and target gene expression was calculated by the 2^−^ΔΔCT method. Genes and corresponding primer sequences used for qRT‐PCR (5′‐3′): GAPDH, F‐GACAGTCAGCCGCATCTTCT, R‐GCGCCCAATACGACCAAATC; IL‐1β, F‐TGCAGGCTTCGAGATGAAC, R‐GGGATTTTGTCGTTGCTTGTC; IL‐6, F‐ACTTCCAGCCAGTTGCCTTCTTG, R‐TGGTCTGTTGTGGGTGGTATCCTC.

#### 4.4.4 Western Blotting

Cellular proteins were extracted with RIPA (Biosharp, BL504A) lysis buffer containing protease and phosphatase inhibitors. Protein concentrations were determined by a BCA assay. Equal amounts of protein were separated by SDS‒PAGE and transferred to PVDF membranes. The membranes were blocked with 5% BSA in TBST, probed with primary antibodies overnight at 4°C, and then with secondary antibodies (HRP‐labeled, Proteintech) for 1 h. The signals were detected with a chemiluminescence system and analyzed with ImageJ. The following antibodies were used: GAPDH (Proteintech, 10494‐1‐AP); Collagen II (Affinity, AF0135); Aggrecan (Proteintech, 13880‐1‐AP); MMP13 (Proteintech, 18165‐1‐AP); NF‐κB p65 (Proteintech, 10745‐1‐AP); Phospho‐NF‐κB p65 (CST, 3033); IKBKB (Proteintech, 15649‐1‐AP).

#### 4.4.5 Cell Viability Assay

CEP cells in the Ctrl, DRG, MS, and DRG+MS groups were seeded in 24‐well plates. At predetermined time points, the medium was replaced with fresh medium containing 10% CCK‐8 (Beyotime, C0037) reagent, and the samples were incubated for 1 h at 37°C. The absorbance was measured at 450 nm in 96‐well plates.

#### 4.4.6 Immunofluorescence

The cells were seeded in six‐well plates, fixed with 4% paraformaldehyde, permeabilized with 0.5% Triton X‐100, blocked with immunofluorescence blocking solution, and incubated with primary antibodies overnight at 4°C. After being washed, the cells were incubated with fluorescent secondary antibodies for 1 h, stained with DAPI (Beyotime, C1002), and observed under a fluorescence microscope. The following antibodies were used: CGRP (Abcam, ab81887); Piezo2(Invitrogen, PA5‐72976); TUJ1(Proteintech, 66375‐1‐Ig).

#### 4.4.7 RNA Sequencing

CEPs were divided into Ctrl, DRG, MS, and DRG+MS groups and cultured for 48 h. Total RNA was extracted with TRIzol reagent, and RNA integrity (RIN ≥8.0) and concentration were assessed. Strand‐specific RNA‐seq libraries were constructed and sequenced on the NovaSeq 6000 platform (Illumina, USA). Bioinformatics analysis was performed on the raw data. Differentially expressed genes were screened (|log2FC| ≥1 and FDR <0.05) and analyzed via GO and KEGG enrichment analysis.

#### 4.4.8 ELISA

Rat CGRP1, IL‐6, and IL‐1β ELISA kits (Elabscience) were used according to the manufacturer's instructions to determine the concentrations of the corresponding factors in the cell supernatants.

#### 4.4.9 Calcium Ion Influx

Primary cultured DRG neurons were loaded with a Fluo‐4 AM (Beyotime, S1061S) calcium fluorescent probe (5 µM, incubated at 37°C for 30 min in the dark), and the excess dye was eluted with Hanks balanced salt solution. The cells were imaged in real time under a fluorescence microscope with an excitation wavelength of 488 nm and an emission wavelength ranging from 500–550 nm. The change in fluorescence intensity (ΔF/F_0_, where F_0_ is the baseline fluorescence value) was calculated using ImageJ software.

#### 4.4.10 Statistical Analysis

The data are expressed as the means ± standard deviations. Intergroup differences were analyzed by an unpaired two‐tailed Student's t test, and differences among multiple groups were analyzed via one‐way ANOVA with Tukey's post hoc test using GraphPad Prism 9.0. *P* < 0.05 was considered to indicate statistical significance.

## Conflicts of Interest

The authors declare no conflicts of interest.

## Supporting information




**Supporting File**: advs75332‐sup‐0001‐SuppMat.docx.

## Data Availability

The data that support the findings of this study are available from the corresponding author upon reasonable request.
