# Supplementary material for: Piezo2 Mediates a Vicious Cycle of “Mechanical Homeostasis Imbalance—Inflammation” in Sensory Nerves and the Cartilage Endplate
Source: Adv Sci (Weinh). 2026 Apr 16;13(38):e07299. doi: 10.1002/advs.202507299 (PMC13335748; doi:10.1002/advs.202507299)
Supplement: Supplementary file 1 — Supporting File: advs75332‐sup‐0001‐SuppMat.docx. [file ADVS-13-e07299-s001.docx]

**Supporting Information**


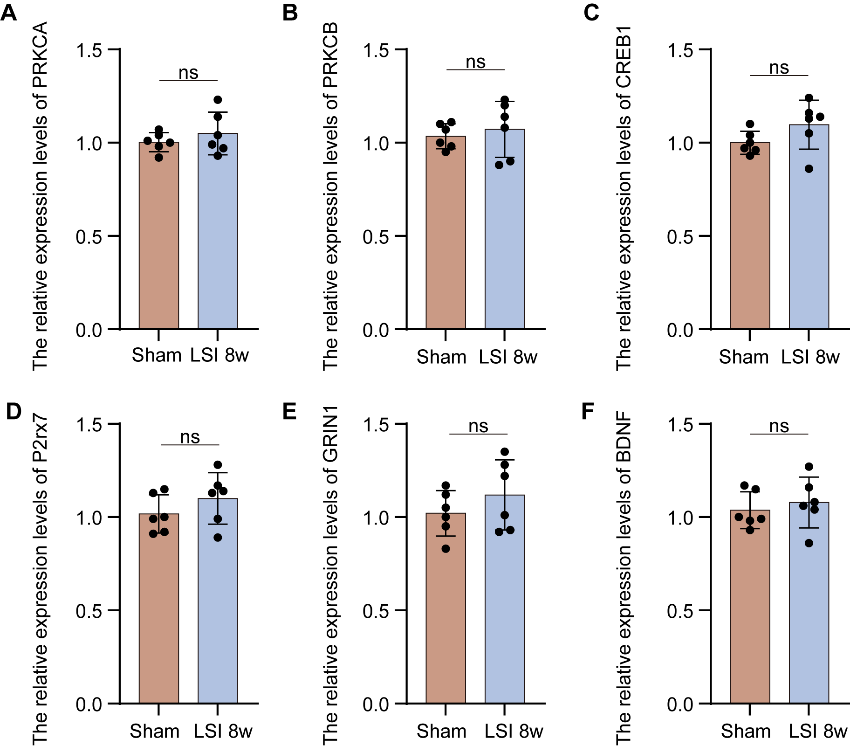


**Figure S1.** PCR of molecules involved in the PKC/cAMP, extracellular ATP, and central sensitization pathways in rat DRG tissue. (A-F) The relative expression levels of mRNAs. Statistical significance was determined by Student’s t test (ns, P≥0.05).


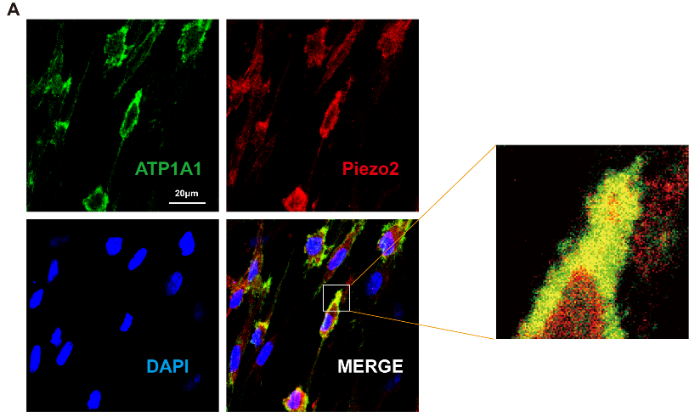


**Figure S2. Representative immunofluorescence images of DRG neurons.** (A) Partial colocalization of membrane proteins ATP1A1 and Piezo2 indicates that Piezo2 is predominantly expressed on the cell membrane.

**
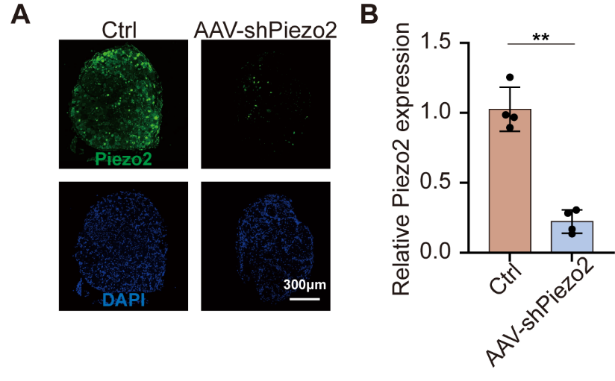
**

**Figure S3. Direct injection of** **AAV-shPiezo2 into the rat DRG to knock down Piezo2.** (A) Piezo2 immunofluorescence and (B) quantification in DRG neurons after Piezo2 knockdown. Statistical significance was determined by ‌Student’s t test‌ (**P < 0.01).


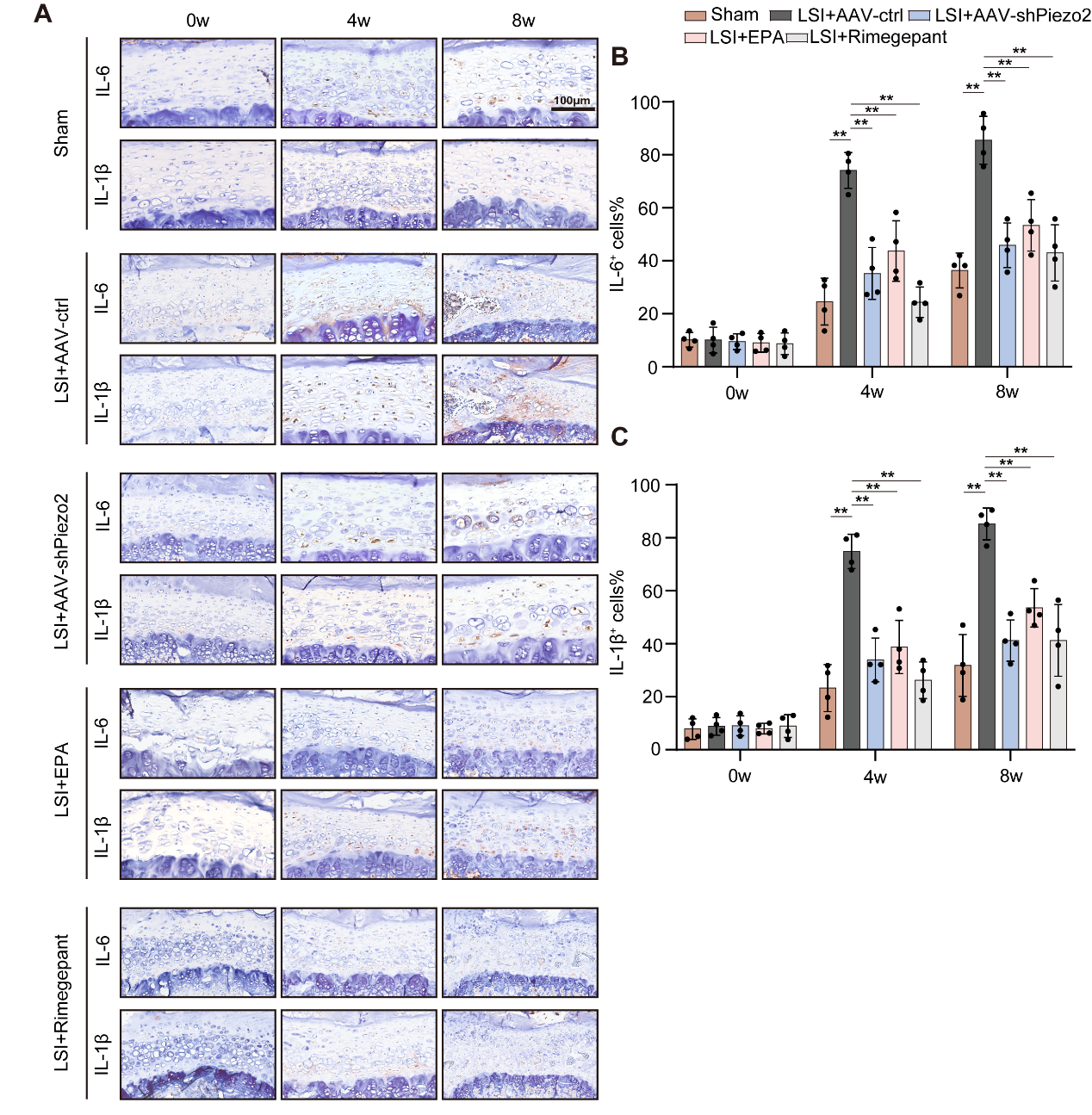


**Figure S4.** **Targeting Piezo2 and CGRP reduces inflammatory factors in LSI models.** (A) Representative IHC staining of caudal L4-L5 CEPs. (B-C) IHC quantification of IL-6 and IL-1β in CEPs. Statistical significance was determined by one-way ANOVA (** P<0.01).


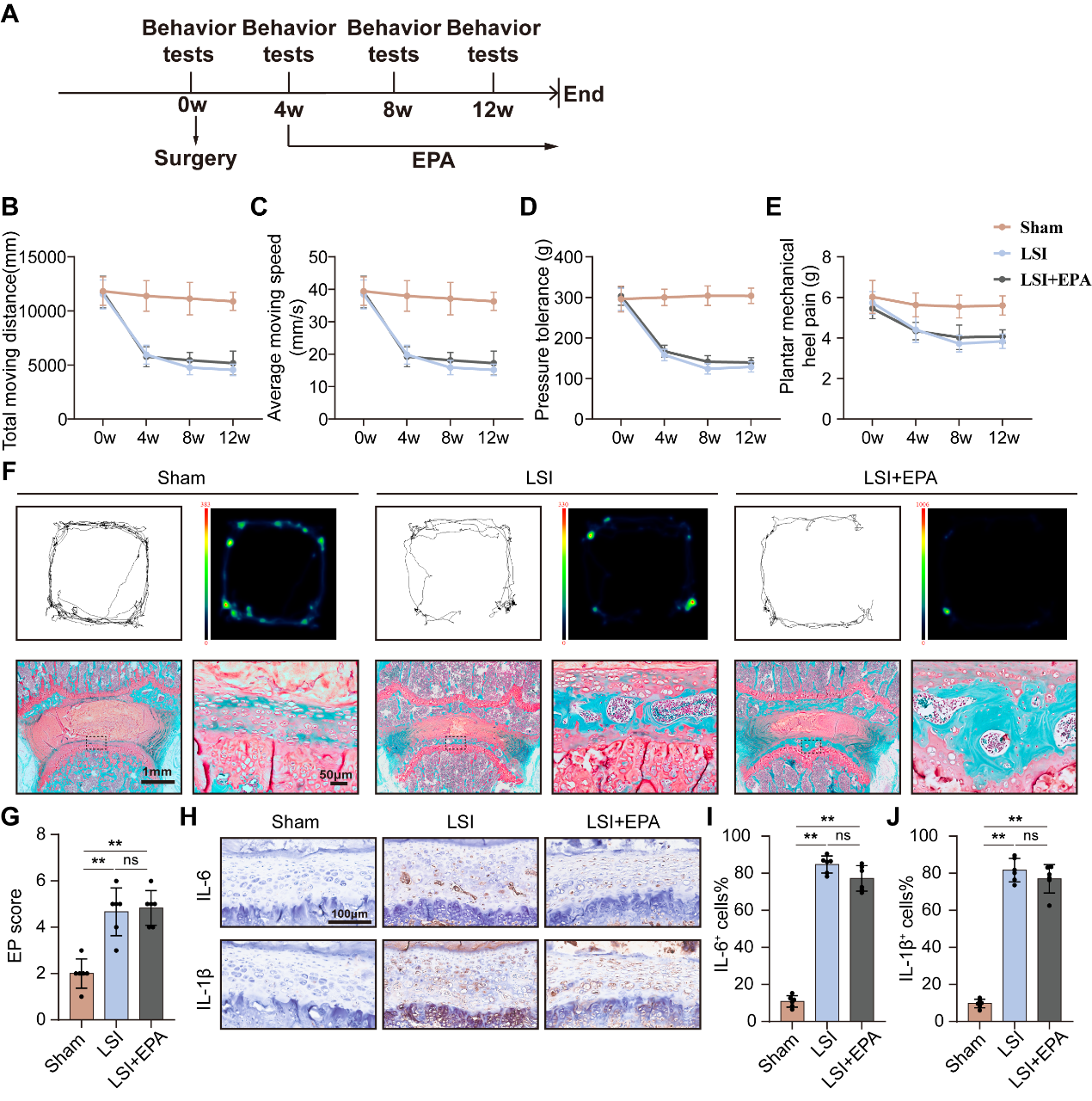


**Figure S5. EPA supplementation at 4 weeks post-LSI surgery fails to improve lumbar pain-related behaviors and cartilage endplate degeneration.** (A) Schematic: Rats exhibit lumbar pain-related behaviors at 4 weeks post-LSI surgery, at which point EPA dietary supplementation is administered. (B) Representative immunofluorescence images of macrophages near the cartilaginous endplates of intervertebral discs in three groups of rats. Quantitative comparisons of behavioral experiments among the three groups of rats: (C) total travel distance and (D) average velocity in the open field test; (E) mechanical pressure tolerance over the L4–L5 dorsal region; and (F) plantar mechanical withdrawal thresholds (n = 6). (G) Upper panel: Open field movement trajectories; lower panel: corresponding representative SO/FG-stained disc sections. (H) Caudal CEP degeneration scores based on panel G. (I) Representative immunohistochemical (IHC) staining of caudal L4-L5 CEPs. (J-K) IHC quantification of IL-6 and IL-1β in CEPs.
